# Impact of New Transport Infrastructure on Walking, Cycling, and Physical Activity

**DOI:** 10.1016/j.amepre.2015.09.021

**Published:** 2016-02

**Authors:** Jenna Panter, Eva Heinen, Roger Mackett, David Ogilvie

**Affiliations:** 1 MRC Epidemiology Unit and UKCRC Centre for Diet and Activity Research (CEDAR), School of Clinical Medicine, University of Cambridge, Cambridge, United Kingdom;; 2 Department of Civil, Environmental and Geomatic Engineering, University College London, London, United Kingdom

## Abstract

**Introduction:**

Walking and cycling bring health and environmental benefits, but there is little robust evidence that changing the built environment promotes these activities in populations. This study evaluated the effects of new transport infrastructure on active commuting and physical activity.

**Study design:**

Quasi-experimental analysis nested within a cohort study.

**Setting/participants:**

Four hundred and sixty-nine adult commuters, recruited through a predominantly workplace-based strategy, who lived within 30 kilometers of Cambridge, United Kingdom and worked in areas of the city to be served by the new transport infrastructure.

**Intervention:**

The Cambridgeshire Guided Busway opened in 2011 and comprised a new bus network and a traffic-free walking and cycling route. Exposure to the intervention was defined using the shortest distance from each participant’s home to the busway.

**Main outcome measures:**

Change in weekly time spent in active commuting between 2009 and 2012, measured by validated 7-day recall instrument. Secondary outcomes were changes in total weekly time spent walking and cycling and in recreational and overall physical activity, measured using the validated Recent Physical Activity Questionnaire. Data were analyzed in 2014.

**Results:**

In multivariable multinomial regression models—adjusted for potential sociodemographic, geographic, health, and workplace confounders; baseline active commuting; and home or work relocation—exposure to the busway was associated with a significantly greater likelihood of an increase in weekly cycle commuting time (relative risk ratio=1.34, 95% CI=1.03, 1.76) and with an increase in overall time spent in active commuting among the least active commuters at baseline (relative risk ratio=1.76, 95% CI=1.16, 2.67). The study found no evidence of changes in recreational or overall physical activity.

**Conclusions:**

Providing new sustainable transport infrastructure was effective in promoting an increase in active commuting. These findings provide new evidence to support reconfiguring transport systems as part of public health improvement strategies.

## Introduction

Physical inactivity is a major contributor to morbidity and mortality, and increasing regular physical activity—particularly among the least active—is likely to improve the health of individuals and populations.^1^, ^2^ However, there is a lack of clear evidence of effective strategies to achieve this.^3^ Public health advocacy increasingly focuses on active travel as a target for intervention, and active commuting offers a comparatively easy way to integrate exercise into daily life.^3^ People who walk or cycle to work or commute by public transport tend to be more physically active, and to have more favorable body composition and cardiovascular risk, than those who do not.^4^, ^5^, ^6^ However, few studies have evaluated the effects of reconfiguring transport systems in favor of active travel, leaving major scientific uncertainty around how the projected health and environmental benefits can be realized in practice.^3^, ^7^

It is often difficult or impossible to evaluate the effects of large-scale changes to the built environment using RCT methods. This calls for the use of quasi-experimental study designs, which present particular challenges in relation to defining exposure, constructing controlled comparisons, and minimizing the impact of residual confounding.^8^ In addition, previous intervention studies in this area have often been limited by insufficient follow-up periods or imprecise measures of the duration or volume of activities, which are important for estimating their health impacts.^7^, ^9^, ^10^, ^11^ Recent guidance illustrates how “natural experiments” can be used to generate more-robust evidence of the effects of environmental changes despite these challenges, and provides a framework for the design and analysis of studies in this area.^8^

This study used quasi-experimental methods to test the hypothesis that exposure to new infrastructure to promote walking, cycling, and public transport—the Cambridgeshire Guided Busway—would result in an increase in time spent walking and cycling on the commute and higher levels of overall physical activity. The secondary aim was to investigate the extent to which these effects differed between population subgroups. A complementary paper describes the broader impacts of the intervention on travel mode share.^12^

## Methods

### The Intervention: the Cambridgeshire Guided Busway

The Cambridgeshire Guided Busway is a major transport infrastructure project comprising a new bus network and an adjacent 22-kilometer traffic-free walking and cycling route in and around Cambridge, described in detail elsewhere (www.thebusway.info).^13^ For much of the route, buses run on a guideway completely segregated from other traffic, but in places—notably for approximately 5 kilometers through the city center—they use the existing road network (Appendix File 1, available online). The path can be accessed at bus stops and other points along the route. Construction began in March 2007, and although completion was scheduled for summer 2009, in fact the busway was opened more than 2 years late on August 7, 2011.

### Study Design, Setting, and Participant Recruitment

The authors evaluated the busway using a quasi-experimental analysis nested within a cohort study of commuters, the Commuting and Health in Cambridge study. The methods for participant recruitment and data collection^13^, ^14^ and baseline findings^15^ have been reported elsewhere. Briefly, participants aged ≥16 years who worked in areas of Cambridge to be served by the busway and lived within approximately 30 kilometers of the city were recruited before the busway was completed, through a predominantly workplace-based strategy (Appendix File 2, available online). The Hertfordshire Research Ethics Committee approved the study and the baseline data collection (reference number 08/H0311/208) and the Cambridge Psychology Research Ethics Committee approved the follow-up data collection (reference number 2014.14). All participants provided written informed consent.

### Data Collection

Participants received a baseline postal questionnaire^15^ between May and October 2009 and annual follow-up questionnaires, matched to the same week of the year, when possible. Because the busway was not opened until August 2011, the 2012 survey was used for the follow-up measure in these analyses (Appendix File 3, available online).

### Measures

At both time points, participants reported all travel modes used on the commute in the last 7 days; if they had walked or cycled any part of their journeys, they also reported the average time spent doing so per trip. Total weekly time spent walking and cycling on the commute was computed and shown to have acceptable validity, with only a small mean overestimation compared with objective measures for walking (2.37 minutes/trip) and cycling (1.12 minutes/trip).^16^ The criteria used to assess commuting data quality, and therefore inclusion in analysis, are given in Appendix File 2 (available online).

Participants completed the Recent Physical Activity Questionnaire (RPAQ), which uses comparatively simple validated measures to assess activities across the intensity spectrum at home, at work, for recreation, and for transport in the last 4 weeks.^17^ The three derived outcomes variables were total weekly time spent: walking and cycling for commuting and recreation, in recreational moderate-to-vigorous physical activity, and in overall physical activity (Appendix File 2, available online).

Descriptive spatial analysis of the sociodemographic and behavioral characteristics of the cohort suggested that comparable intervention and control groups could not be created based crudely on area of residence,^18^ so the authors used an individual measure of proximity to represent exposure to the intervention. It was hypothesized that the intervention could promote walking and cycling either as modes of travel along the path or as feeder modes to the bus service. Using ArcGIS, version 9.1, the distance from each participant’s home to the nearest busway stop or path access point (whichever was closer) was computed using any combination of the road network and traffic-free or informal paths represented in the Ordnance Survey’s Integrated Transport Network and OpenStreetMap. As use of the busway decreased nonlinearly with distance,^18^ exposure was modeled as the square root of the negative of the distance.

Participants reported the characteristics shown in Table 1 at baseline. At follow-up, participants were also asked about any life events, such as pregnancy or changes in caring responsibilities, in the last year.

### Statistical Analysis

All analyses were conducted in 2014. Analyses tested for differences in baseline characteristics between the sample with valid primary outcome data at both time points and the remainder of the baseline sample using *t*-test, chi-squared test, and signed-rank test. Change scores from baseline to follow-up were computed for all outcomes, and scores of >±300 minutes/week in the primary outcome (*n*=9) were truncated to 300. Many participants reported no active commuting at either time point, and the assumptions of linear regression were violated. Therefore, the primary outcome was categorized as “no change,” “increase,” and “decrease,” and the secondary outcomes using tertiles because no participants reported exactly the same quantity of physical activity.

Briefly, multivariable multinomial logistic regression models were used to assess the relationships between exposure to the intervention and outcomes, with progressive adjustment to systematically account for potential confounders. Changes in commuting were also modeled separately for walking and cycling to investigate the extent to which any overall effects were explained by specific behaviors. Models were repeated subsequently, including further adjustment for variables representing other changes in life circumstances. For active commuting and overall physical activity, the authors also tested for interactions with ten hypothesized effect modifiers: baseline activity, sex, age, education, housing tenure, presence of children and availability of a car in the household, urban−rural status, distance to work, and relocation of home or work during the study. Full details are given in Appendix File 2 (available online).

### Sensitivity Analyses

Although the study used outcome measures with good construct and unusually strong criterion validity,^16^, ^17^ the “noise” of measurement error may mask the “signal” of small but important changes in individual behavior. The authors therefore conducted sensitivity analyses. For active commuting, the definition of an effect was limited to a substantial change of ±50 minutes/week (equivalent to a change of at least 5 minutes/trip, assuming a 5-day working week), redefining smaller changes as “no change.” For physical activity, an alternative (“RPAQ+”) measure of overall physical activity was computed using a more detailed measure of active commuting (Appendix File 2, available online).

## Results

Of the 1,143 participants who provided valid data on active commuting at baseline, 469 also provided valid data at follow-up (Table 1, Appendix, Appendix [available online]). Participants were aged between 20 and 71 years at baseline (M=44.3, SD=11.1), 67% were women, and most had at least degree-level education (75%) and at least one car in their household (88%). Those providing valid data at follow-up tended to be older (mean age, 44.3 vs 40.9, *p*=0.001) and more likely to own their home (78.2% vs 69.2%, *p*=0.001) than those who did not. There were no significant differences in other sociodemographic characteristics or active commuting at baseline. Although a direct comparison with the target population is difficult because commuters were recruited from an area not coterminous with administrative boundaries, comparison with census data for residents of Cambridge city and surrounding district council areas aged 16−64 years suggested that the sample contained a higher proportion of women, older adults, and those with a degree, and a smaller proportion of those who rented their home and those aged 16−30 years (Appendix File 4, available online).

**Figure 1:**
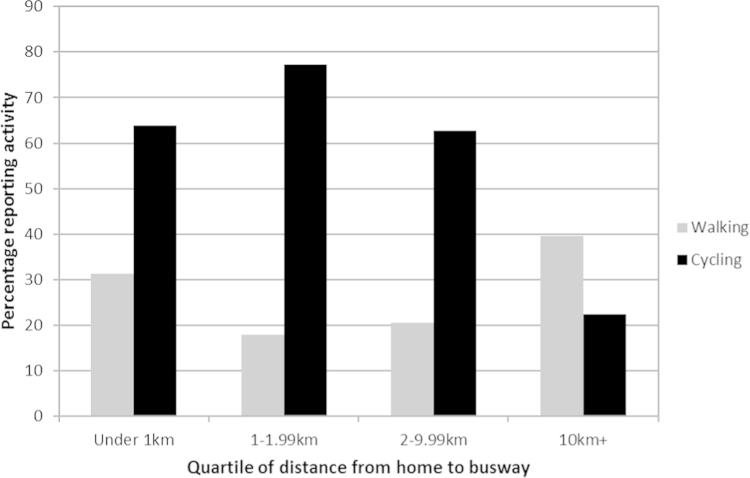
Associations between exposure to the busway and percentage of participants reporting any walking or cycling on the commute at baseline.

*Note:* Quartiles used for graphical presentation only.

Although time spent in overall physical activity changed little over the 3 years, larger changes were observed in the average time spent in active commuting (Appendix File 4, available online). This decreased between baseline and follow-up (median of 120 vs 100 minutes/week, *p*=0.001) and this was mostly explained by a decrease in cycling (median of 70 vs 40 minutes/week, *p*=0.016). Participants living closer to the busway were more likely to report cycling, but less likely to report walking, than those living further away (both *p*<0.05); these associations were not linear (Figure 1).

Unadjusted associations of exposure to the busway with the various outcomes are summarized in Appendix File 4 (available online). In multivariable models adjusted for age and sex, exposure to the busway was associated with changes in total weekly time spent in active commuting, and specifically with changes in time spent cycling (Table 2, Model 1). After further adjustment (Model 3), the effect on total time spent in active commuting was attenuated to the null but the effect on cycling persisted: Exposure to the busway was associated with a significantly greater likelihood of an increase in weekly cycle commuting time (relative risk ratio [RRR]=1.34, 95% CI=1.03, 1.76). This corresponds, for example, to participants living 4 kilometers from the busway being 34% more likely to have increased their cycle commuting time than those living 9 kilometers away. Among those who reported more cycle commuting at follow-up than at baseline, the mean increase was 86.6 (SD=73.9) minutes/week. Further adjustment for other putative confounders changed the results only slightly, with little impact on effect size or statistical significance (Appendix File 4, available online). Sensitivity analysis using the more stringent definition of change of at least 50 minutes/week showed a somewhat stronger effect on cycling (RRR=1.44, 95% CI=1.03, 2.03; Appendix File 4, available online).

Evidence for the effects on total time spent walking and cycling for commuting and recreation was congruent with that for active commuting: The authors found no significant effect on walking and cycling in combination, but a significant effect on total time spent cycling (RRR=1.32, 95% CI=1.04, 1.68, Table 3). No evidence of a significant effect on total time spent in either recreational or overall physical activity was found, including in sensitivity analysis using the RPAQ+ measure (Table 3).

The study found some evidence that the effect of the intervention on active commuting was moderated by baseline active commuting (*p*=0.02 for interaction), with a significant effect on total active commuting time only among those who reported the lowest levels of active commuting at baseline (RRR=1.76, 95% CI=1.16, 2.67). No evidence of differential effects on active commuting was found for any of the other tested population subgroups, or on overall physical activity (all *p*>0.1).

## Discussion

The provision of new infrastructure to promote walking, cycling, and public transport was associated with an increase in time spent cycling on the commute, and with an increase in overall time spent in active commuting among those least active at baseline. These findings are particularly encouraging for several reasons. First, they were observed against a background decrease in active commuting in the cohort. Second, those participants who increased their cycling tended to report sizeable changes, with a mean increase of 80 minutes/week, more than half the recommended weekly “dose” of activity.^1^ Third, sensitivity analysis also suggested that these effects were not just a result of small changes in individual behavior, but reflected substantial increases in time spent cycling. Together with other complementary outcome analyses,^12^ the finding of a stronger overall effect among the least active commuters at baseline supports the hypothesis that the overall increase in cycling reflects new cycle commuters, not merely existing cyclists making more or longer trips. Finally, the increases in active commuting were not offset by a compensatory decrease in recreational physical activity. Epidemiologic studies suggest the beneficial effects of active commuting on cardiovascular risk independent of other physical activities,^6^ and modeling studies suggest that the health benefits of a population shift toward active travel would greatly outweigh the disbenefits.^19^ Three systematic reviews^7^, ^9^, ^10^ have identified few rigorous studies of environmental or policy approaches to shifting population active travel patterns and have called for more evidence, particularly in respect of effects on the volume of activity and equity impacts, including whether interventions are effective in promoting walking and cycling among the least active. Although high-quality studies in this area remain few in number, the present findings, together with those of epidemiologic and modeling studies, provide increasing empirical support for the argument that reconfiguring transport systems to support active travel will improve population health. Further natural experimental studies of interventions in a wider range of contexts would help strengthen the evidence to support more generalizable causal inference and intersectoral policymaking to improve health.

Although the physical and social conditions for cycling in Cambridge are more favorable than those in much of the rest of the United Kingdom and may have contributed to the results reported here, the quality of the infrastructure is patchy and well below that of other European countries, such as Belgium, Denmark, or the Netherlands.^20^ It is therefore important to have shown that improving transport infrastructure can foster change, while acknowledging that a supportive social environment may also be a prerequisite.^21^ Complementary qualitative analysis has shown that perceptions of the busway’s attributes and its portrayal in the media were important in shaping behavioral responses.^22^, ^23^ More importantly, the analysis of effect modification found a significant effect among those who were least active at baseline. This suggests that environmental changes of this kind have the potential to shift the population distribution of activity, rather than merely enabling those who are already active to do a little more.^24^

This study found no evidence of effects on time spent walking on the commute or in overall physical activity. The lack of an effect on walking may reflect the facts that the path was less frequently used for walking than for cycling,^18^ and walking to work was less prevalent than cycling to work^15^; the difficulty of capturing incidental walking^16^; or people’s interactions with the spatial, environmental, and economic conditions of the study area.^13^, ^15^, ^18^ The lack of an effect on overall physical activity is consistent with those of other studies of environmental changes, even when the new or improved environments were well received and used.^25^ Estimates of overall physical activity derived from self-report measures are subject to large measurement error and the present results do not exclude the possibility of a false-negative finding.^16^, ^26^ The increases in active commuting were not offset by a compensatory decrease in recreational physical activity, and changes in overall physical activity may take longer to emerge as new habits develop over time.^27^

Drawing on Medical Research Council guidance on natural experimental studies and recommendations from the National Institute for Health and Care Excellence,^8^, ^28^ the authors prespecified the hypotheses and assessed outcomes using changes within individuals over time using measures that were validated and specific to the nature of the intervention.^13^, ^16^ The graded measure of exposure served as a basis for controlled comparisons, and allowed more accurate reflection of the environmental changes experienced by each participant, while avoiding misclassification of their exposure.^8^ The findings are consistent with those of other studies that used a similar approach to analysis.^25^, ^27^ This study also systematically considered and accounted for a series of confounders representing plausible alternative explanations, providing a considerable increase in rigor over most previous studies.^7^, ^11^, ^20^, ^28^ Though a quasi-experimental study design cannot completely isolate the effect of an intervention from that of other measured or unmeasured confounders, the strengths of this study give greater confidence that the inferences are consistent with a causal interpretation.^10^

### Limitations

This study had a comparatively low retention rate, but this is not unusual in natural experimental studies of public health interventions.^28^ Although there was no evidence of attrition bias with respect to the primary outcome, the composition and attrition of our cohort do somewhat limit the generalizability of the findings. Though the sample had levels of car ownership comparable with that of England and Wales as a whole,^29^ women and graduates were over-represented in the sample of mostly healthy commuters, and Cambridge has an established cycling culture. The sample also reported higher levels of physical activity than those of respondents from the east of England in the 2008 Health Survey for England, but this is at least partly attributable to major differences in measurement; another study using RPAQ reported comparable findings.^30^ Though the results may not be directly generalizable to other populations, they are important in demonstrating the potential for change in a setting where cycling is seen as acceptable, and the sample of predominantly healthy, car-owning, middle-aged commuters represents a key target population for the prevention of chronic noncommunicable diseases.^4^

## Conclusions

This study has shown that providing new sustainable transport infrastructure was effective in promoting an increase in active commuting, particularly cycling. However, further research is needed to provide more-robust and generalizable evidence to support policymaking to help shift population physical activity patterns. This should aim to establish the effectiveness of a range of environmental and policy intervention strategies, understand how their effects come about, and the likely cardiometabolic impacts of active travel and its promotion.

## Figures and Tables

**Table 1:** Characteristics of Baseline and Follow-up Samples at Entry to the Study

**Characteristics**	**Baseline (*****n*****=1,143)**	**Follow-up**
**(*****n*****=469)**
Demographic characteristics		
Age (years, M [SD])	42.3 (11.4)	44.3 (11.1)
Sex		
Male	360 (31.5)	158 (33.5)
Female	783 (68.5)	311 (66.5)
Any child in the household		
No	913 (80.0)	340 (72.5)
Yes	229 (20.0)	129 (27.5)
Socioeconomic characteristics		
Education		
Less than degree-level education	319 (28.1)	120 (25.2)
Degree-level education	817 (71.9)	349 (74.8)
Housing tenure		
Renting or other	310 (27.2)	104 (21.8)
Owner-occupier	829 (72.8)	365 (78.2)
Car ownership		
No car	169 (14.8)	56 (12.1)
One car	517 (45.2)	224 (47.8)
Two or more cars	457 (40.0)	188 (40.1)
Geographic characteristics		
Urban-rural status		
Urban	752 (65.8)	316 (67.3)
Town and fringe	221 (19.4)	80 (17.1)
Village and hamlet	169 (14.8)	73 (15.6)
Workplace characteristics		
Distance from home to work		
Self-reported distance (km, median [IQR])	8.0 (3.2−20.9)	8.0 (4.0−20.9)
Provision of workplace car parking		
No parking	366 (32.3)	151 (32.4)
Free parking	420 (37.1)	172 (36.9)
Paid parking	347 (30.6)	143 (30.7)
Health characteristics		
Weight status		
Underweight or normal weight	707 (62.8)	304 (66.1)
Overweight or obese	418 (37.2)	165 (33.9)
Health condition^a^		
No	1,302 (91.5)	428 (91.6)
Yes	121 (8.5)	39 (8.4)
Active weekly commuting, minutes, median (IQR)		
Spent in active commuting	115.5 (0−200)	120 (33−200)
Spent walking on the commute	0 (0−40)	0 (0−20)
Spent cycling on the commute	70 (0−160)	80 (0−160)

*Note: n* (%) unless otherwise specified. All characteristics were assessed at baseline. Not all subcolumns sum to 1,143 or 469 owing to missing data in covariates. Self-reported height and weight were used to compute BMI and assign participants to WHO categories of weight status. In the United Kingdom, degrees are awarded by a university after completion of undergraduate courses.

a Limiting long-term illness or difficulty walking quarter of a mile on the flat. IQR, interquartile range.

**Table 2:** Associations Between Exposure to the Busway and Changes in Time Spent in Active Commuting

**Outcome behavior**	***n***	**Change in min/week, M (SD)**^a^	**RRR (95% CI)**^b^		
**Model 1**^c^	**Model 2**^d^	**Model 3**^e^
Active commuting	454				
No change	122	0 (0)	Ref		
Increase	136	80.7 (70.9)	1.31 (1.11, 1.56)^**^	1.14 (0.90, 1.45)	1.14 (0.90, 1.46)
Decrease	196	−81.8 (69.0)	1.34 (1.14, 1.57)^**^	1.06 (0.83, 1.37)	1.07 (0.83, 1.37)
Walking on the commute	456				
No change	297	0 (0)	Ref		
Increase	76	73.4 (66.6)	0.84 (0.71, 1.00)	0.90 (0.69, 1.19)	0.90 (0.69, 1.18)
Decrease	83	−84.7 (70.8)	0.90 (0.76, 1.07)	1.13 (0.83, 1.55)	1.13 (0.83, 1.55)
Cycling on the commute	468				
No change	214	0 (0)	Ref		
Increase	108	86.6 (74.0)	1.66 (1.37, 2.03)^**^	1.34 (1.03, 1.76)^*^	1.34 (1.03, 1.76)^*^
Decrease	146	−85.9 (67.6)	1.53 (1.30, 1.81)^**^	1.00 (0.73, 1.36)	1.00 (0.73, 1.37)

*Note:* Boldface indicates statistical significance (^*^p<0.05; ^**^p<0.001).

a Mean change (standard deviation) in the relevant outcome variable in each outcome category.

b Adjusted relative risk ratios (RRR) and 95% CIs for a change in weekly duration of the given behavior per unit of proximity (square root of distance) to busway.

c Model 1 is adjusted for age and sex.

d Model 2 is adjusted for variables in model 1 plus baseline education, car ownership, home ownership, children in the household, health condition, BMI, urban-rural status, distance to work, workplace car parking provision and baseline value of the outcome for the model in question.

e Model 3 is adjusted for variables in model 2 plus any change in home or work location. min, minutes.

**Table 3:** Associations Between Exposure to the Busway and Changes in Total Time Spent Walking and Cycling and in Recreational and Overall Physical Activity

**Outcome behavior**	***n***	**Change in min/week, M (SD)**^a^	**RRR (95% CI)**^b^		
**Model 1**^c^	**Model 2**^d^	**Model 3**^e^
Total walking and cycling	469				
Mid tertile (~no change)	156	−7.7 (28.7)	Ref		
Top tertile (~increase)	156	223.9 (264.2)	1.11 (0.95, 1.30)	1.21 (0.96, 1.52)	1.21 (0.96, 1.52)
Bottom tertile (~decrease)	157	−246.1 (297)	1.02 (0.88, 1.19)	1.17 (0.92, 1.47)	1.17 (0.92, 1.47)
Total walking					
Mid tertile (~no change)	158	2.4 (20.6)	Ref		
Top tertile (~increase)	157	179.9 (213.6)	1.01 (0.86, 1.18)	1.00 (0.80, 1.25)	1.00 (0.80, 1.25)
Bottom tertile (~decrease)	154	−188.3 (256.8)	0.99 (0.85, 1.16)	0.93 (0.74, 1.18)	0.94 (0.74, 1.18)
Total cycling					
Mid tertile (~no change)	168	−1.5 (4.5)	Ref		
Top tertile (~increase)	158	113.5 (151)	1.65 (1.38, 1.96)^**^	1.32 (1.04, 1.68)^*^	1.32 (1.04, 1.68)^*^
Bottom tertile (~decrease)	143	−123.3 (184)	1.56 (1.33, 1.84)^**^	1.20 (0.94, 1.53)	1.20 (0.94, 1.54)
Total recreational physical activity					
Mid tertile (~no change)	157	−5.5 (41.2)	Ref		
Top tertile (~increase)	156	323.4 (455.0)	0.95 (0.81, 1.12)	0.98 (0.78, 1.22)	0.98 (0.78, 1.22)
Bottom tertile (~decrease)	156	−370.16 (439.0)	0.95 (0.81, 1.12)	0.88 (0.69, 1.13)	0.88 (0.69, 1.13)
Total physical activity (RPAQ)					
Mid tertile (~no change)	156	−14.5 (51.4)	Ref		
Top tertile (~increase)	157	390.4 (475.7)	1.02 (0.88, 1.20)	1.06 (0.85, 1.32)	1.06 (0.84, 1.32)
Bottom tertile (~decrease)	156	−494.6 (596.4)	1.09 (0.93, 1.28)	1.00 (0.77, 1.29)	1.00 (0.77, 1.29)
Total physical activity (RPAQ+)					
Mid tertile (~small decrease)	156	−105.4 (54.9)	Ref		
Top tertile (~no change/small increase)	156	265.9 (453.6)	1.00 (0.86, 1.17)	0.93 (0.75, 1.17)	0.93 (0.74, 1.17)
Bottom tertile (~large decrease)	157	−655.5 (691.6)	1.06 (0.91, 1.24)	0.94 (0.72, 1.24)	0.94 (0.72, 1.23)

*Note:* Boldface indicates statistical significance (^*^p<0.05; ^**^p<0.001).

a Mean change (standard deviation) in the relevant outcome variable in each outcome category.

b Adjusted RRRs and 95% CIs for a change in weekly duration of the given behavior per unit of proximity (square root of distance) to busway.

c Model 1 is adjusted for age and sex.

d Model 2 is adjusted for variables in model 1 plus baseline education, car ownership, home ownership, children in the household, health condition, BMI, urban-rural status, distance to work, workplace car parking provision, and baseline value of the outcome for the model in question.

e Model 3 is adjusted for variables in model 2 plus any change in home or work location. min, minutes; RAPQ, Recent Physical Activity Questionnaire; RRR, relative risk ratio.
