# Supplementary material for: Impact of New Transport Infrastructure on Walking, Cycling, and Physical Activity
Source: Am J Prev Med. 2016 Feb;50(2):e45–53. doi: 10.1016/j.amepre.2015.09.021 (PMC4712020; doi:10.1016/j.amepre.2015.09.021)
Supplement: Supplementary file 1 — Supplementary data [file mmc1.pdf]

Appendix File 1. Additional information on the intervention.

The Cambridgeshire Guided Busway

(a) Map of the busway

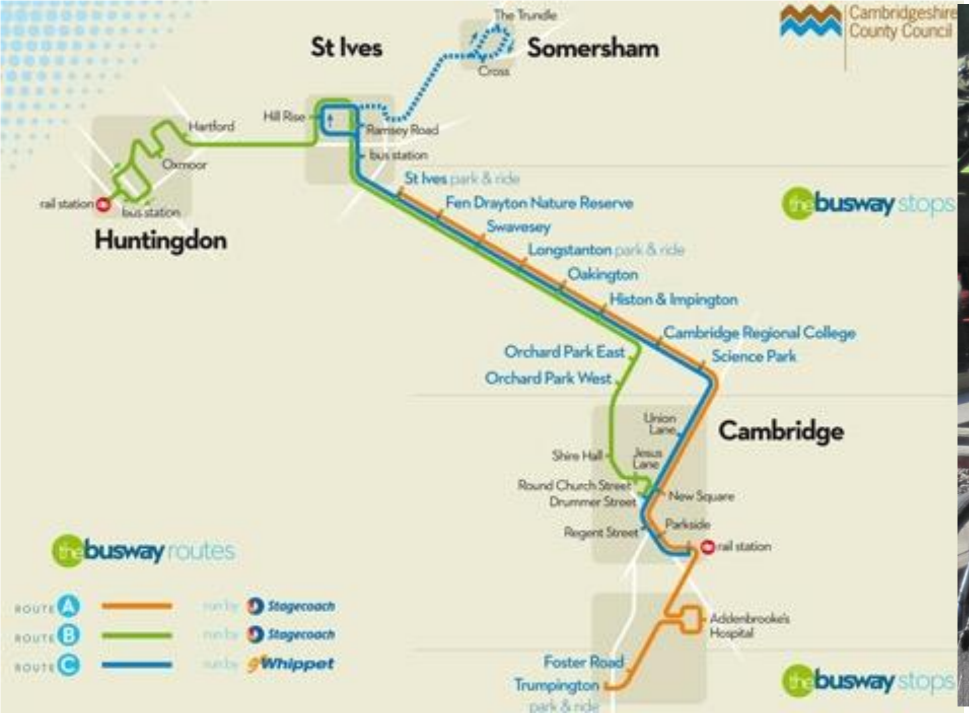

(b) Traffic-free path for walking and cycling

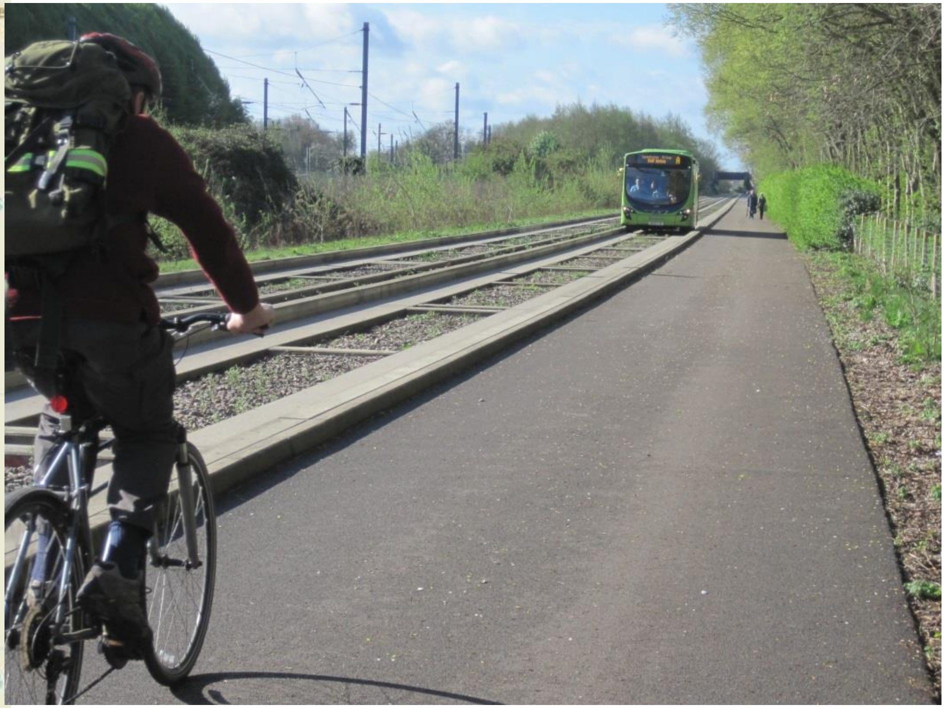

Map reproduced with kind permission of Cambridgeshire County Council. Photo © Eva Heinen and reproduced with permission.

Appendix File 2

Additional Methodological Information

Recruitment

To avoid breaching data protection legislation and to assure participants of the study's independence, commuters were not recruited using employer-based sampling frames such as staff databases, but were invited to opt in to the study through a variety of strategies including recruitment stands, advertisements and emails distributed through corporate mailing lists. A variety of workplaces contributed to participant recruitment. These included local authorities, healthcare providers, retail outlets and institutions of higher and further education distributed across a range of city centre and urban fringe locations in Cambridge. Those taking part in any concurrent physical activity study were excluded.

Criteria for Validity of Primary Outcome Data

We defined participants with valid data on active commuting as those who reported working on at least three days of the week, and reported their mode(s) of travel to or from work on at least three days of the week, at both time points. We attempted to validate missing data by cross-checking the number of working hours in the last week (reported elsewhere in the questionnaire) and any other explanations provided by participants. This ensured that part-time workers and those who reported no trips on a given day (for example as a result of sickness or holiday) were not unnecessarily excluded from analysis unless their data were truly missing.

Derivation of Secondary Outcome Measures

The Recent Physical Activity Questionnaire (RPAQ) uses comparatively simple validated measures to assess activities across the intensity spectrum at home, at work, for recreation and for transport in the last 4 weeks. Home-based physical activity was estimated using measures of the frequency of stair climbing on a typical weekday and weekend day and of the duration of sedentary behaviors such as TV viewing on a typical weekday and weekend day. Physical activity at work was estimated using a categorical measure of the predominant intensity of activity at work and the number of hours worked per week. The reported frequencies of each recreational activity were divided by four to give a weekly estimate, multiplied by the average duration of each session of activity and summed to give an estimate of the weekly time spent in recreational activity. Physical activity for transport was estimated using information on the frequency with which different modes of travel were used for commuting (reported on an ordinal scale from 'never' to 'always'), the number of trips per week and the self-reported commute distance. The calculation of time spent in MVPA already incorporated correction for over-reporting and we therefore applied no further truncation to the secondary outcomes.

We converted the measures of activity in each domain to a common metric of minutes per week and used this information to derive three secondary outcome measures. Total weekly time spent walking and cycling for commuting and recreation was derived by summing the time spent walking or cycling for recreation (reported in RPAQ) and the time spent in active commuting described above. Total weekly time spent in recreational moderate-to-vigorous physical activity (MVPA) was derived by summing the time spent in all recreational activities reported in RPAQ which were classified as ≥3 times metabolic equivalent (3 METs) according to the physical activity compendium. Total weekly time spent in overall physical activity was derived by summing the time spent in activities above the MVPA

intensity threshold across the four domains (home, work, recreation and transport) reported in RPAQ.

Sensitivity Analysis Using RPAQ+

Of all the activity domains within RPAQ, transport was the one with the lowest criterion validity in the original RPAQ validation study. We computed an alternative ('RPAQ+') measure of overall physical activity using our more detailed measure of active commuting as a substitute for the cruder RPAQ measure in the transport domain. All of the other measures of activity from the work, home and recreation domains remained the same in this RPAQ+ calculation.

Specification of Multivariable Regression Models

We used progressive multivariable adjustment for different sets of covariates to systematically account for potential confounders and thereby assess key plausible alternative explanations for the outcomes observed. We first adjusted for age and sex (Model 1) and then for baseline education, car ownership, home ownership, children in the household, health condition, BMI, urban-rural status, distance to work, workplace car parking provision and baseline value of the outcome for the model in question (Model 2). These have been shown to be associated with active commuting and changes in active commuting, both in this study and in the wider literature,[1-4] and preliminary analysis showed that many of these baseline characteristics were not evenly distributed in our sample. For example, younger and more mobile participants tended to live closer to the busway before it was completed. At this stage we also adjusted for each participant's baseline level of the behavioral outcome for the model in question, to account for initial differences in activity levels. We then considered changes in participants' life circumstances over the period of observation. We first adjusted for a binary

indicator of whether a participant had moved home or work during the study, because relocation often prompts a change in travel behavior[5] and moving might also have changed a participant's exposure to the intervention (Model 3). We excluded the 20 cases with missing data for any of the covariates from models 2 and 3, and did not impute any missing data on outcomes or covariates. To account for as many other potential explanations as possible, we repeated our models including further adjustment for variables representing other changes in life circumstances, namely car and home ownership, household composition, workplace car parking provision and health conditions. However, the case for treating these changes as confounders was less clear and they could also plausibly be considered as causes, consequences or moderators of behavior change in these analyses. All analysis was undertaken in Stata 13.1.

Assessment of Effect Modification

In the maximally adjusted models (Model 3), we also tested for interactions with ten characteristics that we hypothesized could modify the effects of the intervention by influencing the propensity to change behavior. Six of these (sex, age and highest educational qualification of the participant; and housing tenure, presence of children and availability of a car in the household) were entered using the categorical or continuous measures shown in Table 1. The other four were collapsed into categorical measures as follows. Urban-rural status was categorized as urban/town or fringe/village/hamlet; distance to work was categorized as <5km, 5-15km or ≥15km; and whether a participant had moved home or work during the study was categorized as yes or no. For the baseline activity was it was categorized as zero, 1-149 or ≥150 min/week for cycling; zero and 1> min/week for walking, tertiles for active commuting (<35, 35 to 149.9 and ≥150 min/week) and for the overall physical activity

model, baseline activity was dichotomized using the median value (≤422 min/week or ≥423 min/week).

Sample Size

In our original study design we aimed to achieve a sample of 788 participants, which would have provided 80% power to detect a standardized mean difference between intervention and control groups at one-year follow-up of 0.2, equivalent to a mean increase of 2 min/day in our primary outcome of time spent in active commuting.[6] The delayed completion of the busway resulted in an enforced follow-up period of 3 years rather than one year and a concomitant reduction in the final sample size for analysis. This smaller sample would have provided 80% power to detect a mean difference of 4 min/day, which although larger than the original target effect size is still commensurate with the effect sizes observed for comparable interventions in previous studies.[7,8] However, the original sample size calculation was never intended to give more than a broad indication, particularly given the need to model an outcome that was not normally distributed and to adjust for multiple covariates.

References

1. Panter J, Griffin S, Dalton AM, Ogilvie D. Patterns and predictors of changes in active commuting over 12 months. *Prev Med.* 2013;57(6):776-784. http://dx.doi.org/10.1016/j.ypmed.2013.07.020.
2. Panter J, Griffin S, Jones A, Mackett R, Ogilvie D. Correlates of walking and cycling to work: baseline results from the Commuting and Health in Cambridge study. *Int J Behav Nutr Phys Act.* 2011;8:124. http://dx.doi.org/10.1186/1479-5868-8-124.
3. Panter J, Griffin S, Ogilvie D. Active commuting and perceptions of the route environment: A longitudinal analysis. *Prev Med.* 2014;67:134-140. http://dx.doi.org/10.1016/j.ypmed.2014.06.033.
4. Panter JR, Jones AP. Attitudes and the environment: What do and don't we know? *J Phys Act Health.* 2010;7(4):551-561.
5. Jones C, Ogilvie D. Motivations for active commuting: a qualitative investigation of the period of home or work relocation. *Int J Behav Nutr Phys Act.* 2012;9:109. http://dx.doi.org/10.1186/1479-5868-9-109.
6. Ogilvie D, Griffin S, Jones A, Mackett R, Guell C, Panter J, et al. Commuting and health in Cambridge: a study of a 'natural experiment' in the provision of new transport infrastructure. *BMC Public Health.* 2010;10(1):703. http://dx.doi.org/10.1186/1471-2458-10-703.
7. Goodman A, Sahlqvist S, Ogilvie D. New Walking and Cycling Routes and Increased Physical Activity: One- and 2-Year Findings From the UK iConnect Study. *Am J Public Health.* 2014;104 (9):e38-46. http://dx.doi.org/10.2105/AJPH.2014.302059.
8. Ogilvie D, Foster C, Rothnie H, et al. Interventions to promote walking: systematic review. *BMJ.* 2007;334:1204-1207. http://dx.doi.org/10.1136/bmj.39198.722720.BE.

Appendix File 3: Follow-up questionnaire

MRC | Epidemiology Unit

Commuting and health in Cambridge Questionnaire 2012

About this questionnaire

This questionnaire booklet has two parts.

Part 1 is a **Recent Physical Activity Questionnaire**. This is designed to find out about your physical activity in your everyday life during the last four weeks. It is divided into three sections:

- Section A asks about your physical activity patterns in and around the house
- Section B asks about your travel to work and your activity at work
- Section C asks about your recreational activities.

Part 2 is a **travel and general questionnaire**. This is designed to find out about your general health, your travel patterns, your views, and some background information about yourself.

YOUR ANSWERS WILL BE TREATED AS STRICTLY CONFIDENTIAL

How to complete the questionnaire

The questionnaire should take about 20 minutes to complete. Please try to answer every question. Please use a blue or black pen.

Some questions ask you to **tick** a box. Please tick the box that applies to you.

Example: Are you male or female? Male ☑ Female ☐

Other questions ask you to **write numbers** in a box.

Example: What is your age? *Write in* [53] years

Don't worry if you make a **mistake** — just cross out the mistake and put in the correct answer.

Example: Do you have access to a bicycle? Yes ☑ No ☑

PART 1: RECENT PHYSICAL ACTIVITY QUESTIONNAIRE

Section A — Home activities

Getting about

1 **Which form of transport have you used most often in the last four weeks apart from your journey to and from work?**

Tick one only

- Car / motor vehicle ☐
- Walking ☐
- Public transport ☐
- Cycling ☐

TV, DVD or video viewing

Average over the last four weeks

2 **Hours of TV, DVD or video watched per day**

Tick one box on each line

	None	Less than 1 hour a day	1 to 2 hours a day	2 to 3 hours a day	3 to 4 hours a day	More than 4 hours a day
On a weekday before 6 pm	☐	☐	☐	☐	☐	☐
On a weekday after 6 pm	☐	☐	☐	☐	☐	☐
On a weekend day before 6 pm	☐	☐	☐	☐	☐	☐
On a weekend day after 6 pm	☐	☐	☐	☐	☐	☐

Computer use at home but not at work (e.g. internet, email, Playstation, Xbox, Gameboy, etc.)

Average over the last four weeks

3 **Hours of home computer use per day**

Tick one box on each line

	None	Less than 1 hour a day	1 to 2 hours a day	2 to 3 hours a day	3 to 4 hours a day	More than 4 hours a day
On a weekday before 6 pm	☐	☐	☐	☐	☐	☐
On a weekday after 6 pm	☐	☐	☐	☐	☐	☐
On a weekend day before 6 pm	☐	☐	☐	☐	☐	☐
On a weekend day after 6 pm	☐	☐	☐	☐	☐	☐

Stair climbing at home

Average over the last four weeks

4 Number of times you climbed up a flight of stairs (approx. 10 steps) each day at home

Tick one box on each line	None	1 to 5 times a day	6–10 times a day	11–15 times a day	16–20 times a day	More than 20 times a day
On a weekday	☐	☐	☐	☐	☐	☐
On a weekend day	☐	☐	☐	☐	☐	☐

Section B — Activity at work

Please answer this section to describe if you have been in paid employment at any time during the last four weeks or you have done regular, organised voluntary work.

5 Have you been in employment during the last four weeks?

Tick one only Yes ☐ No ☐

6 During the last four weeks how many hours work did you do per week?

Write in number
If none, write '0'

Four weeks ago	☐
Three weeks ago	☐
Two weeks ago	☐
Last week	☐

Type of work

7 We would like to know the type and amount of physical activity involved in your work. Please tick the option that best corresponds with your occupation(s) in the last four weeks from the following four possibilities:

Tick one only

Sedentary occupation
You spend most of your time sitting (such as in an office) ☐

Standing occupation
You spend most of your time standing or walking. However, your work does not require intense physical effort (e.g. shop assistant, hairdresser, guard) ☐

Manual work
This involves some physical effort including handling of heavy objects and use of tools (e.g. plumber, electrician, carpenter) ☐

Heavy manual work
This implies very vigorous physical activity including handling of very heavy objects (e.g. dock worker, miner, bricklayer, construction worker) ☐

Travel to and from work in the last four weeks

8 What is the approximate distance from your home to your work?

Write in ☐☐☐ miles **OR** ☐☐☐ km

9 How many times a week did you travel from home to your main work? Count outward journeys only. *Write in number If none, write '0'* ☐

10 How did you normally travel to work?

Tick one box on each line	Always	Usually	Occasionally	Never or rarely
By car or motor vehicle	☐	☐	☐	☐
By works or public transport	☐	☐	☐	☐
By bicycle	☐	☐	☐	☐
Walking	☐	☐	☐	☐

Please give the full postal address and postcode of your main place of work DURING THE LAST FOUR WEEKS

11 Postal address ☐

12 Postcode ☐☐☐☐☐☐☐

Please give the full postal address and postcode of your home

13 Postal address

14 Postcode ☐☐☐☐☐☐☐

Section C — Recreation

The following questions ask about how you spent your leisure time.

Please indicate how often you did each activity on average over the last four weeks

Please indicate the average length of time that you spent doing the activity on each occasion.

Example: If you went walking for pleasure for 40 minutes once a week, and if you did weeding or pruning every fortnight and took 1 hour and 10 minutes on each occasion, you would complete the table below as follows:

Please give an answer for the NUMBER OF TIMES you did the following activities in the past four weeks and the AVERAGE TIME you spent on each activity

	**Number of times you did the activity in the last four weeks**							**Average time per episode**	
Please complete EACH line	None	Once in the last 4 weeks	2 to 3 times in the last 4 weeks	Once a week	2 to 3 times a week	4 to 5 times a week	Every day	Hours	Mins
Weeding and pruning			✓					1	10
Walking for pleasure				✓					40

Now complete the table on pages 6 and 7

15 Please give an answer for the NUMBER OF TIMES you did the following activities in the past four weeks and the AVERAGE TIME you spent on each activity

	Number of times you did the activity in the last four weeks							Average time per episode	
Please complete EACH line	None	Once in the last 4 weeks	2 to 3 times in the last 4 weeks	Once a week	2 to 3 times a week	4 to 5 times a week	Every day	Hours	Mins
Swimming — competitive									
Swimming — leisurely									
Backpacking or mountain climbing									
Walking for pleasure (not as a means of transport)									
Racing or rough terrain cycling									
Cycling for pleasure (not as a means of transport)									
Mowing the lawn									
Watering the lawn or garden									
Digging, shovelling or chopping wood									
Weeding or pruning									
DIY, e.g. carpentry, home or car maintenance									
High impact aerobics or step aerobics									
Other types of aerobics									
Exercise with weights									
Conditioning exercises, e.g. using a bike or rowing machine									
Floor exercises, e.g. stretching, bending, keep fit or yoga									
Dancing, e.g. ballroom or disco									

Please give an answer for the NUMBER OF TIMES you did the following activities in the past four weeks and the AVERAGE TIME you spent on each activity

	**Number of times you did the activity in the last four weeks**							**Average time per episode**	
Please complete EACH line	None	Once in the last 4 weeks	2 to 3 times in the last 4 weeks	Once a week	2 to 3 times a week	4 to 5 times a week	Every day	Hours	Mins
Competitive running									
Jogging									
Bowling — indoor, lawn or ten pin									
Tennis or badminton									
Squash									
Table tennis									
Golf									
Football, rugby or hockey									
Cricket									
Rowing									
Netball, volleyball or basketball									
Fishing									
Horse-riding									
Snooker, billiards or darts									
Musical instrument playing or singing									
Ice skating									
Sailing, wind-surfing or boating									
Martial arts, boxing or wrestling									

PART 2: TRAVEL AND GENERAL QUESTIONNAIRE

About your health

16 **Do you have any long-term illness, health problem or disability which limits your daily activities or the work you can do?** Include problems which are due to old age.

Tick one only Yes ☐ No ☐

17 **Do you have any difficulty walking for a quarter of a mile on the level?**

Tick one only Yes ☐ No ☐

18 **In the PAST TWELVE MONTHS how many days were you off sick for health reasons?** *Write in number If none, write '0'* ☐

19 **How tall are you?** (with your shoes off)

Write in ☐ ft ☐ in **OR** ☐ cm

20 **How much do you weigh?** (in light indoor clothes)

Write in ☐ st ☐ lb **OR** ☐ kg

The next section asks for your views about your health. This information will help keep track of how you feel and how well you are able to do your usual activities. For each of the following questions, please tick the one box that best describes your answer.

21 **Overall, how would you rate your health during the PAST FOUR WEEKS?**

Excellent	Very good	Good	Fair	Poor	Very poor
☐	☐	☐	☐	☐	☐

22 **During the PAST FOUR WEEKS, how much did physical health problems limit your usual physical activities (such as walking or climbing stairs)?**

Not at all	Very little	Somewhat	Quite a lot	Could not do physical activities
☐	☐	☐	☐	☐

23 **During the PAST FOUR WEEKS, how much difficulty did you have doing your daily work, both at home and away from home, because of your physical health?**

None at all	A little bit	Some	Quite a lot	Could not do daily work
☐	☐	☐	☐	☐

24 How much BODILY pain have you had during the PAST FOUR WEEKS?

None	Very mild	Mild	Moderate	Severe	Very severe
☐	☐	☐	☐	☐	☐

25 During the PAST FOUR WEEKS, how much energy did you have?

Very much	Quite a lot	Some	A little	None
☐	☐	☐	☐	☐

26 During the PAST FOUR WEEKS, how much did your physical health or emotional problems limit your usual social activities with family or friends?

Not at all	Very little	Somewhat	Quite a lot	Could not do social activities
☐	☐	☐	☐	☐

27 During the PAST FOUR WEEKS, how much have you been bothered by emotional problems (such as feeling anxious, depressed or irritable)?

Not at all	Slightly	Moderately	Quite a lot	Extremely
☐	☐	☐	☐	☐

28 During the PAST FOUR WEEKS, how much did personal or emotional problems keep you from doing your usual work, school or other daily activities?

Not at all	Very little	Somewhat	Quite a lot	Could not do daily activities
☐	☐	☐	☐	☐

SF-8™ 4-Week Recall Version — © 1999-2001 — QualityMetric, Inc. — All rights reserved

29 How would you describe yourself? ***Tick one only***

I am a current smoker ☐
I am an ex-smoker ☐
I have never smoked ☐

30 How often do you have a drink containing alcohol? ***Tick one only***

Never ☐
Monthly or less ☐
2 to 4 times a month ☐
2 to 3 times a week ☐
4 or more times a week ☐

31 How many drinks containing alcohol do you have on a typical day when you are drinking? *Tick one only*

- **1 to 2** ☐
- **3 to 4** ☐
- **5 to 6** ☐
- **7, 8 or 9** ☐
- **10 or more** ☐

32
Have you been injured in a road accident in the PAST THREE YEARS? Please include incidents where you were in a vehicle, on a bicycle or motorbike, or a pedestrian.

Tick one only Yes ☐ → ***Go to Q. 33*** No ☐ → ***Go to Q. 35***

33 Thinking about the most recent incident in which you were injured, were you..? *Tick one only*

- The driver of a vehicle ☐
- A passenger in a car or van ☐
- A passenger on public transport ☐
- A motorcyclist ☐
- A cyclist ☐
- A pedestrian ☐

34 Did you receive any medical attention as a result of your injuries at any time following the incident? *Tick all that apply*

- No medical attention received ☐
- Yes – first aid at the roadside ☐
- Yes – at a doctor's surgery or minor injuries unit ☐
- Yes – at a hospital Accident & Emergency department ☐
- Yes – as an inpatient staying overnight in hospital ☐

About your travel options

35
How many cars or vans are owned, or available for use, by members of your household?
Do not include motorcycles, scooters or mopeds.

Write in number
If none, write '0' ☐

36 Do you hold a full driving licence valid in Great Britain either to drive a car or to drive a motorcycle, scooter or moped?

Tick one only Yes ☐ No ☐

37 **Do you have access to car parking at your place of work?** This includes parking anywhere on the site, for example in a multi-storey car park. It does not include parking on the streets nearby or at a park-and-ride.

Tick one only

Yes, and I have to pay to park there ☐
Yes, and I do not have to pay to park there ☐
No ☐

38 **Do you ever travel by car for part or all of the journey to or from work?**
This includes as a passenger in a car driven by someone else.

Tick one only Yes ☐ → ***Go to Q. 39*** No ☐ → ***Go to Q. 42***

39 **When you use a car for the journey to or from work, is the car usually parked at a park-and-ride site?**

Tick one only Yes ☐ No ☐

Thinking about the car you are most likely to use to travel to and from work:

40 **What type of fuel does the car use?**

Tick one only

Petrol ☐
Diesel ☐
Hybrid or other ☐

41 **What is the engine size of the car?**

Write in ☐ cc **OR** ☐ litres

42 **Do you have access to a bicycle?**

Tick one only Yes ☐ No ☐

43 **Do you know how to ride a bicycle?**

Tick one only Yes ☐ → ***Go to Q.44*** No ☐ → ***Go to Q.48***

44 **How old were you when you learned to ride a bicycle?** *Write in age* ☐ years

45 **How old were you when you first used a bicycle regularly to get to places?**

Write in age ☐ years **OR** *Tick* ☐ I have never used a bicycle to get to places

46 **Do you ever cycle part or all of the journey to or from work?**

This includes cycling to or from a bus stop, railway station or park-and-ride.

Tick one only Yes ☐ → ***Go to Q. 47*** No ☐ → ***Go to Q. 48***

47 How long does the cycling part of the journey usually take? ☐ minutes each way

48
Do you ever walk part or all of the journey to or from work?
This includes walking to or from a bus stop, railway station or park-and-ride.

Tick one only Yes ☐ → ***Go to Q. 49*** No ☐ → ***Go to Q. 50***

49 How long does the walking part of the journey usually take? ☐ minutes each way

About your travel to and from work in the last seven days

In this section, we are interested in **how you travelled to and from work on each of the last seven days**.

50 **For each of the last seven days, please tell us what time you started and finished work and tick all the modes of transport you used on the journey to and from work.** If you did not travel to work on a particular day (either because it was a day off or because you worked at home), please tick the box 'Did not travel to work'. If your journey to and from work was the same on more than one day, you can tick the box 'Same as previous' instead of repeating the information again. *We have given you an example for one day in the first row of the table.*

Which modes of transport did you use on this journey? *Tick all that apply*

Day of the week	Time started work	Time finished work	Did not travel to work		Same as previous	Guided bus	Other bus or coach	Train or underground	Car, taxi or van	Motorcycle or moped	Bicycle	Walking	Other
Thu	7.30 am/~~pm~~	3.30 ~~am~~/pm	☐	**To work**		☑	☐	☐	☐	☐	☐	☑	☐
				**From work**		☑	☐	☐	☐	☐	☐	☑	☐
	am/pm	am/pm	☐	**To work**		☐	☐	☐	☐	☐	☐	☐	☐
				**From work**		☐	☐	☐	☐	☐	☐	☐	☐
	am/pm	am/pm	☐	**To work**	☐	☐	☐	☐	☐	☐	☐	☐	☐
				**From work**	☐	☐	☐	☐	☐	☐	☐	☐	☐
	am/pm	am/pm	☐	**To work**	☐	☐	☐	☐	☐	☐	☐	☐	☐
				**From work**	☐	☐	☐	☐	☐	☐	☐	☐	☐
	am/pm	am/pm	☐	**To work**	☐	☐	☐	☐	☐	☐	☐	☐	☐
				**From work**	☐	☐	☐	☐	☐	☐	☐	☐	☐
	am/pm	am/pm	☐	**To work**	☐	☐	☐	☐	☐	☐	☐	☐	☐
				**From work**	☐	☐	☐	☐	☐	☐	☐	☐	☐
	am/pm	am/pm	☐	**To work**	☐	☐	☐	☐	☐	☐	☐	☐	☐
				**From work**	☐	☐	☐	☐	☐	☐	☐	☐	☐

About all the journeys you made yesterday

In this section, we are interested in more detail about **all the journeys you made yesterday** (between 3 a.m. yesterday and 3 a.m. today).

51 **Please list each journey you made yesterday to get from place to place.** These might include, for example, going to work, going out to get lunch, coming home from work, going shopping, going to the doctor's, visiting friends, or escorting someone else (e.g. taking a child to school). Please include time spent travelling on foot or by bike, even if this was part of a longer journey (e.g. by bus or train). But please **do not include** journeys you made as part of your job (e.g. as a delivery driver), or walking or cycling purely for recreation or exercise (e.g. walking the dog).

*We have given you an example of **one** journey. This person walked for ten minutes to the bus stop, rode on the bus for 22 minutes, and then walked for five minutes to get to work (a total of 15 minutes walking). They did not count the time spent waiting for the bus.*

	Office use only	**What was the purpose of the journey?** Please give a simple description, e.g. 'to get home from work', 'shopping', 'take child to school'	**How many MINUTES did you spend TRAVELLING by each mode of transport on this journey?** Do not count time spent waiting for buses, trains etc.							
			Guided bus	Other bus or coach	Train or underground	Car, taxi or van	Motorcycle or moped	Bicycle	Walking	Other
	☐	From home to work	22	22					15	
	☐	From work to home	22	22					15	
**Journey 1**	☐									
**Journey 2**	☐									
**Journey 3**	☐									

Continue over the page if necessary

About all the journeys you made yesterday (continued)

How many MINUTES did you spend TRAVELLING by each mode of transport on this journey?
Do not count time spent waiting for buses, trains etc.

	Office use only	**What was the purpose of the journey?** Please give a simple description, e.g. 'to work', 'to get home from work', 'shopping', 'take child to school'	Guided bus	Other bus or coach	Train or underground	Car, taxi or van	Motorcycle or moped	Bicycle	Walking	Other
**Journey 4**										
**Journey 5**										
**Journey 6**										
**Journey 7**										
**Journey 8**										
**Journey 9**										
**Journey 10**										

About your workplace

Tick one per row

52 Does your workplace have the following?

	Yes	No	Don't know
Parking stands or racks for bicycles	☐	☐	☐
Somewhere to store waterproof clothing or cycle helmets	☐	☐	☐
Changing rooms	☐	☐	☐
Adult cycle training	☐	☐	☐
A 'bicycle users' group	☐	☐	☐
A 'walk to work' or 'bike to work' day or week	☐	☐	☐
A cycle to work scheme *(An employer scheme to loan bicycles and cyclists' safety equipment to employees as a tax-free benefit, e.g. CycleScheme)*	☐	☐	☐
A travel plan *(A formal document which identifies ways of encouraging more employees to walk, cycle or use public transport to get to work)*	☐	☐	☐

For each of the following statements, please tick one box to show how strongly you agree or disagree.

Tick one per row

53 At my workplace:

	Strongly agree	Agree	Neither agree nor disagree	Disagree	Strongly disagree
Many of my colleagues **WALK** all or part of the way to and from work	☐	☐	☐	☐	☐
Many of my colleagues **CYCLE** all or part of the way to and from work	☐	☐	☐	☐	☐
Many of my colleagues **DRIVE** to and from work	☐	☐	☐	☐	☐
Members of senior management **WALK OR CYCLE** all or part of the way to and from work	☐	☐	☐	☐	☐
Members of senior management **DRIVE** to and from work	☐	☐	☐	☐	☐

About your views on travelling to and from work

For each of the following statements about your journey to and from work, please tick one box to show how strongly you agree or disagree.

Tick one per row

**54 On my journey to and from work:**	Strongly agree	Agree	Neither agree nor disagree	Disagree	Strongly disagree
It is pleasant to walk	☐	☐	☐	☐	☐
The roads are dangerous for cyclists	☐	☐	☐	☐	☐
There is convenient public transport	☐	☐	☐	☐	☐
There are convenient routes for cycling	☐	☐	☐	☐	☐
There is little traffic	☐	☐	☐	☐	☐
There are no convenient routes for walking	☐	☐	☐	☐	☐
It is safe to cross the road	☐	☐	☐	☐	☐
**55 For me, to get to and from work next time:**					
Overall, it would be good to **USE A CAR**	☐	☐	☐	☐	☐
Most people who are important to me would support my **USING A CAR**	☐	☐	☐	☐	☐
It would be easy for me to **USE A CAR**	☐	☐	☐	☐	☐
I intend to **USE A CAR**	☐	☐	☐	☐	☐
It would be pleasant to **USE A CAR**	☐	☐	☐	☐	☐
Most people who are important to me think I should **USE A CAR**	☐	☐	☐	☐	☐
I would be able to **USE A CAR**	☐	☐	☐	☐	☐
I am likely to **USE A CAR**	☐	☐	☐	☐	☐

For each of the following statements about your journey to and from work, please tick one box to show how strongly you agree or disagree.

Tick one per row

56 **USING A CAR to get to and from work is something:**	Strongly agree	Agree	Neither agree nor disagree	Disagree	Strongly disagree
I do frequently	☐	☐	☐	☐	☐
I do automatically	☐	☐	☐	☐	☐
that would require effort not to do	☐	☐	☐	☐	☐
that belongs to my daily routine	☐	☐	☐	☐	☐
I would find hard not to do	☐	☐	☐	☐	☐
that's typically 'me'	☐	☐	☐	☐	☐
I have been doing for a long time	☐	☐	☐	☐	☐
57 **For me, to get to and from work next time:**					
Overall, it would be good to **WALK**	☐	☐	☐	☐	☐
Most people who are important to me would support me **WALKING**	☐	☐	☐	☐	☐
It would be easy for me to **WALK**	☐	☐	☐	☐	☐
I intend to **WALK**	☐	☐	☐	☐	☐
It would be pleasant to **WALK**	☐	☐	☐	☐	☐
Most people who are important to me think I should **WALK**	☐	☐	☐	☐	☐
I would be able to **WALK**	☐	☐	☐	☐	☐
I am likely to **WALK**	☐	☐	☐	☐	☐

For each of the following statements about your journey to and from work, please tick one box to show how strongly you agree or disagree.

Tick one per row

58 For me, to get to and from work next time:	Strongly agree	Agree	Neither agree nor disagree	Disagree	Strongly disagree
Overall, it would be good to **CYCLE**	☐	☐	☐	☐	☐
Most people who are important to me would support me **CYCLING**	☐	☐	☐	☐	☐
It would be easy for me to **CYCLE**	☐	☐	☐	☐	☐
I intend to **CYCLE**	☐	☐	☐	☐	☐
It would be pleasant to **CYCLE**	☐	☐	☐	☐	☐
Most people who are important to me think I should **CYCLE**	☐	☐	☐	☐	☐
I would be able to **CYCLE**	☐	☐	☐	☐	☐
I am likely to **CYCLE**	☐	☐	☐	☐	☐

About your views on bus travel

59 Overall, how would you rate the quality of local bus services?

Very good	Fairly good	Neither good nor poor	Fairly poor	Very poor
☐	☐	☐	☐	☐

The Cambridgeshire Guided Busway is a new transport project in the Cambridge area.

60
Had you previously heard of the Cambridgeshire Guided Busway?

Tick one only Yes ☐ → ***Go to Q.61*** No ☐ → ***Go to Q. 70***

61
Have you travelled on a guided bus in Cambridgeshire?

Tick one only Yes ☐ → ***Go to Q. 63*** No ☐ → ***Go to Q. 62***

62 What are your reasons for not using the guided bus service?
Please give up to three reasons.

☐

If you have not used the guided bus service, please go to Q. 67

63 How many and what types of journey have you made using the guided bus service in the last month?

If you have used the guided bus service for these trips in the last month, how would you have made them if there were no guided bus service?
Tick all that apply

	Number of trips in the last month	By bus or coach	By car	On foot	By bicycle	By another mode of transport	I would not have made this type of trip
Shopping		☐	☐	☐	☐	☐	☐
To or from work		☐	☐	☐	☐	☐	☐
To or from school, college or university (including accompanying children)		☐	☐	☐	☐	☐	☐
On business		☐	☐	☐	☐	☐	☐
Visiting friends or relatives		☐	☐	☐	☐	☐	☐
On personal business (e.g. to the dentist)		☐	☐	☐	☐	☐	☐
On holiday, days out or other leisure trips		☐	☐	☐	☐	☐	☐
Other		☐	☐	☐	☐	☐	☐

64 What do you LIKE about the guided bus service? Please give up to three answers.

65 What do you DISLIKE about the guided bus service? Please give up to three answers.

66 How has use of the guided bus service affected the AMOUNT you walk, cycle or use the car or bus? *Please tick the appropriate box for each mode of transport.*

**I now …**	**MORE** than before	The **SAME** as before	**LESS** than before
Use the bus (including the guided bus)	☐	☐	☐
Use the car	☐	☐	☐
Walk	☐	☐	☐
Cycle	☐	☐	☐

If you have answered '**MORE** than before' or '**LESS** than before' for any of the modes of transport, please briefly state the main reasons why.

☐
☐
☐

67 Have you walked or cycled along any part of the footpath or cycle path beside the guided busway? *Tick all that apply*

Yes – I have walked beside the busway ☐
Yes – I have cycled beside the busway ☐
No – I have not walked or cycled along the paths beside the busway at all ☐

If you have not used the footpath or cycle path, please go to Q. 69

68 How has use of the footpath or cycle path affected the AMOUNT you walk, cycle or use the car or bus? *Please tick the appropriate box for each mode of transport.*

**I now …**	**MORE** than before	The **SAME** as before	**LESS** than before
Use the bus (including the guided bus)	☐	☐	☐
Use the car	☐	☐	☐
Walk	☐	☐	☐
Cycle	☐	☐	☐

If you have answered '**MORE** than before' or '**LESS** than before' for any of the modes of transport, please briefly state the main reasons why.

☐
☐
☐

69 For each of the following statements, please tick one box to show how strongly you agree or disagree.

Tick one per row

**The guided busway has improved:**	Strongly agree	Agree	Neither agree nor disagree	Disagree	Strongly disagree
The range of transport options available	☐	☐	☐	☐	☐
The regularity of transport services	☐	☐	☐	☐	☐
The reliability of transport services	☐	☐	☐	☐	☐
Access to local services	☐	☐	☐	☐	☐
Local air quality	☐	☐	☐	☐	☐
Noise in the local area	☐	☐	☐	☐	☐
The quality of footpaths and cycle paths	☐	☐	☐	☐	☐
The behaviour of other passengers	☐	☐	☐	☐	☐
Your personal safety when travelling	☐	☐	☐	☐	☐
The cost of travel	☐	☐	☐	☐	☐

About your personal views

70 For each of the following statements, please tick one box to show how strongly you agree or disagree.

Tick one per row

	Strongly agree	Agree	Neither agree nor disagree	Disagree	Strongly disagree
I am trying to use the car less for environmental reasons	☐	☐	☐	☐	☐
Unless I can get to a leisure destination by car, I would not go at all	☐	☐	☐	☐	☐
Reducing my car use will not make a difference to congestion problems because most people will not reduce theirs	☐	☐	☐	☐	☐
There are many problems and difficulties with using public transport	☐	☐	☐	☐	☐
I would be willing to pay higher taxes for car use if I knew the revenue would be used to improve public transport	☐	☐	☐	☐	☐
It is important to build more roads to reduce congestion	☐	☐	☐	☐	☐
Being environmentally responsible is important to me as a person	☐	☐	☐	☐	☐
The balance of nature is very delicate and easy to upset by human activities	☐	☐	☐	☐	☐
Environmental threats such as global warming and deforestation have been over exaggerated	☐	☐	☐	☐	☐
I often buy organic food	☐	☐	☐	☐	☐
I often attend meetings organised by an environmental group or charity	☐	☐	☐	☐	☐

About you and your household

71 Are you a student in full time education?

Tick one only Yes ☐ No ☐

72 How long have you lived in the Cambridge area?

EITHER *tick* All my life ☐ **OR** *write in* Since the age of ☐ years

73 How many other people live in your household?
We mean people who have your accommodation as their only or main residence, and who either share at least one meal a day with you or share the living accommodation (living room or sitting room) with you.

Write in number
If none write 0

Children aged under 5 ☐

Children aged between 5 and 15 ☐

Adults aged 16 and over (do not include yourself) ☐

74
Does your household own or rent its accommodation? *Tick one only*

Rents it from the council, a housing association, or a charity ☐

Rents it from a private landlord or letting agency ☐

Partly owns it and partly rents it (shared ownership) ☐

Owns it (including buying with a mortgage) ☐

Other ☐

We are interested in any changes in your household circumstances that may have affected where and when you travel.

75 Have any of the following occurred IN THE LAST YEAR? *Tick all that apply*

You or your partner are now expecting a baby ☐

You or your partner have had a baby ☐

You have been promoted or taken on significant extra responsibilities at work ☐

One or more of your children have started school or moved to a different school ☐

You have become a carer for a family member ☐

Your household income has increased ☐

Your household income has decreased ☐

Other (please specify) ☐

76 On a scale of 1 to 10, how important was the cost of housing in determining your choice of neighbourhood to live in?
Please circle a number

Did not consider cost at all									Cost was the only consideration
1	2	3	4	5	6	7	8	9	10

77 Please think back to what kind of neighbourhood you were hoping to find when you moved to your current address and rank up to three of the considerations below in order of preference.

For example, if low crime was most important, score this (1), followed by local schools (2) and housing quality (3)

	Rank up to three factors
Low crime	☐
Access to shops and services	☐
Visual characteristics of the neighbourhood	☐
Access to public transport links (trains, buses)	☐
Access to main roads	☐
Length of commute for yourself	☐
Length of commute for other adult(s) in household	☐
Housing quality	☐
Characteristics of neighbourhood residents	☐
Local schools	☐
Familiarity with neighbourhood	☐
Child's commute to school	☐
Near family or friends	☐
Other (please specify)	☐

Finally

78 Please enter today's date. *Write in* ☐ (*day of the week*) ☐ (*date*) / ☐ (*month*) / 12

THANK YOU VERY MUCH FOR TAKING PART IN THIS STUDY

Appendix File 4: Additional results

Appendix Figure 1: Flow of participants into and through the study over the three years.

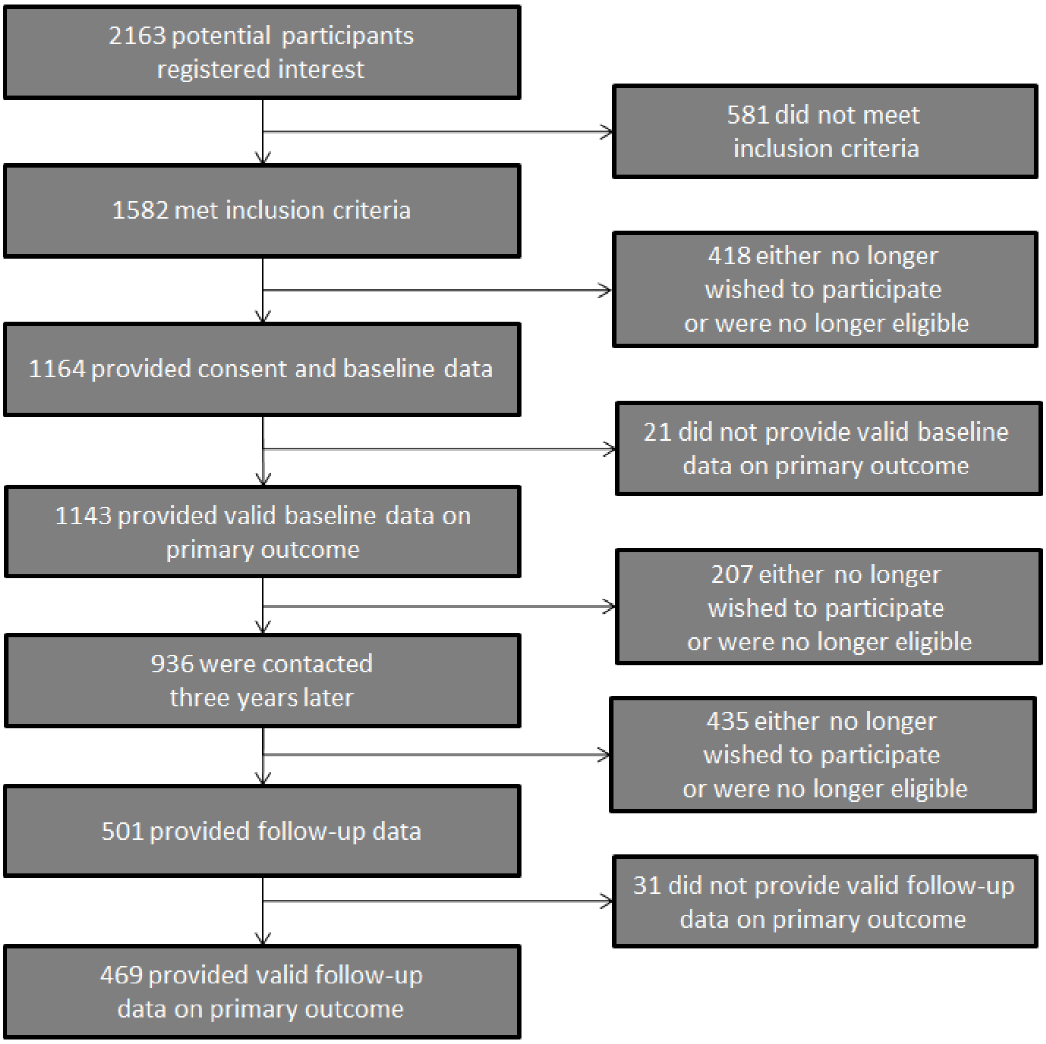

Although a direct comparison with the target population is difficult because we recruited commuters from a heterogeneous area that was not coterminous with administrative boundaries, comparison with census data for residents of Cambridge city and surrounding district council areas aged 16-64 suggested that our sample contained a higher proportion of women, older adults and those with a degree, and a smaller proportion of those who rented their home and those aged 16-30 (Appendix Table 1).

Appendix Table 1. Comparison of Baseline Characteristics of Study Population with Local Resident Population

Characteristic	Prevalence in study sample in 2009	Prevalence in local resident population aged 16-64	Reference population
**Demographic**			
Female	66%	48%	Cambridge City
		50%	South Cambridgeshire
		50%	East Cambridgeshire
Aged 16-30	6%	43%	Cambridge City
		16%	South Cambridgeshire
		15%	East Cambridgeshire
**Socio-economic**			
Degree level or equivalent education	75%	50%	Cambridge City
		44%	South Cambridgeshire
		32%	East Cambridgeshire
Living in rented housing[a]	22%	55%	Cambridge City
		27%	South Cambridgeshire
		28%	East Cambridgeshire
Access to a car	85%	73%	Cambridge City
		95%	South Cambridgeshire
		94%	East Cambridgeshire

[a]Aged 15-64. Source: Census (2011) Standard Area Statistics (England and Wales) (http://www.neighbourhood.statistics.gov.uk). The local authority areas of South Cambridgeshire and East Cambridgeshire represent the majority of the surrounding rural area from which the cohort was drawn.

Appendix Table 2. Distribution of Active Commuting and Physical Activity at Baseline and Follow-up

Activity	% (n) reporting any activity at baseline	Time spent in activity (min/week) Median (IQR)		
		Baseline	Follow-up	*p*-value
**Active commuting**	77.6 (364)	120 (33, 200)	100 (0,170)	0.001
Walking on the commute	27.8 (131)	0 (0, 20)	0 (0, 25)	0.487
Cycling on the commute	56.6 (266)	70 (0,150)	40 (0,150)	0.016
**Walking and cycling for recreation**	83.3 (391)	75 (28, 150)	79 (30, 180)	0.640
Walking for recreation	78.0 (366)	57 (15, 135)	60 (0, 150)	0.551
Cycling for recreation	32.6 (153)	0 (0, 22.5)	0 (0, 19)	0.416
**Total walking and cycling**	95.7 (449)	207 (120, 332)	200 (110, 340)	0.261
Total walking	83.2 (390)	75 (30, 203)	100 (30, 180)	0.630
Total cycling	65.0 (305)	90 (0, 180)	73 (0, 169)	0.064
**Total recreational physical activity**	99.3 (466)	282 (150, 532)	279 (146, 480)	0.282
**Total physical activity**	100 (469)	423 (232, 675)	407 (240, 631)	0.117

IQR, interquartile range; p for differences between baseline and follow-up using a Wilcoxon signed-rank test.

Appendix Table 3. Exposure to the Busway According to the Outcome Categories

Outcome	Distance to busway (km)<br>Median (IQR)
**Change in active commuting**	
No change	4.35 (1.36, 17.90)
Increase	1.72 (1.03, 8.15)
Decrease	1.87 (1.05, 6.00)
**Change in walking on the commute**	
No change	2.00 (1.13, 8.00)
Increase	3.17 (1.07, 14.07)
Decrease	2.01 (0.93, 14.44)
**Change in cycling on the commute**	
No change	5.42 (1.27, 8.14)
Increase	1.55 (0.94, 3.57)
Decrease	1.72 (1.02, 8.14)
**Change in overall physical activity**	
Mid tertile (~no change)	2.38 (1.10, 8.20)
Top tertile (~increase)	2.76 (1.07, 8.63)
Bottom tertile (~decrease)	1.88 (1.10, 8.20)

Appendix Table 4. Associations Between Exposure to the Busway and Changes in Time Spent in Active Commuting After Additional Adjustment for Changes in Sociodemographic and Health Characteristics

Change in active commuting (min/week)	RRR (95% CI)
**Active commuting**	
No change	*Ref*
Increase	1.22 (0.94, 1.57) n.s.
Decrease	1.09 (0.83, 1.43) n.s.
**Walking on the commute**	
No change	*Ref*
Increase	0.96 (0.72, 1.28) n.s.
Decrease	1.20 (0.86, 1.70) n.s.
**Cycling on the commute**	
No change	*Ref*
Increase	**1.37 (1.03, 1.81)**
Decrease	0.98 (0.70, 1.38) n.s.

RRR, relative risk ratio; n.s., not significant
Boldface indicates statistical significance ($p<0.05$).
Model is adjusted for variables in Model 3, Table 3 plus changes in car ownership, home ownership, number of children in the household, workplace car parking provision, and health conditions.

Appendix Table 5. Sensitivity analysis of associations between exposure to the busway and changes in time spent active commuting

Change in walking and cycling on the commute (mins/week)	N	Mean (SD)	RRR (95% CI) Model 1	Model 2	Model 3
**Active commuting**	454				
No change (<50 min/week)	249	-2.1 (21.6)	*Ref*		
Increase (>50 min/week)	81	116.9 (71.4)	**1.26 (1.04, 1.53) ***	1.27 (0.97, 1.66) n.s.	1.27 (0.98, 1.66) n.s.
Decrease (>50 min/week)	124	-112.9 (69.3)	**1.20 (1.02, 1.40) ***	1.20 (0.92, 1.58) n.s.	1.21 (0.92, 1.58) n.s.
**Walking on the commute**	456				
No change (<50 min/week)	362	0.1 (12.6)	*Ref*		
Increase (>50 min/week)	42	111.7 (68)	0.95 (0.76, 1.18) n.s.	0.98 (0.69, 1.39) n.s.	0.98 (0.70, 1.39) n.s.
Decrease (>50 min/week)	52	-118.9 (69.1)	0.98 (0.80, 1.20) n.s.	1.46 (0.99, 2.13) n.s.	1.46 (0.99, 2.16) n.s.
**Cycling on the commute**	468				
No change (<50 min/week)	299	-0.9 (16.3)	*Ref*		
Increase (>50 min/week)	69	120.1 (73.4)	**1.74 (1.34, 2.26) *****	**1.44 (1.03, 2.03)** **	**1.44 (1.03, 2.03) ***
Decrease (>50 min/week)	100	-111.9 (66.9)	**1.38 (1.15, 1.65) *****	0.99 (0.69, 1.40) n.s.	0.99 (0.70, 1.41) n.s.

Notes: Adjusted relative risk ratios and 95% CIs for a change in weekly duration of the given behavior per unit of proximity (square root of distance) to busway.
Boldface indicates statistical significance (p<0.05) *p<0.05, **p<0.01, ***p<0.001.
N, number of participants in each outcome category; Mean (SD), mean and SD for change in the relevant outcome variable in each outcome category; RRR, relative risk ratio; n.s., not significant.
Model 1 is adjusted for age and sex.
Model 2 is adjusted for variables in model 1 plus baseline education, car ownership, home ownership, children in the household, health condition, BMI, urban-rural status, distance to work, workplace car parking provision and baseline value of the outcome for the model in question.
Model 3 is adjusted for variables in model 2 plus any change in home or work location.
